# A Qualitative Exploration of Workarounds Related to the Implementation of National Electronic Health Records in Early Adopter Mental Health Hospitals

**DOI:** 10.1371/journal.pone.0077669

**Published:** 2014-01-16

**Authors:** Gloria Ser, Ann Robertson, Aziz Sheikh

**Affiliations:** 1 eHealth Research Group, Centre for Population Health Sciences, The University of Edinburgh, Edinburgh, United Kingdom; 2 Division of General Internal Medicine and Primary Care, Brigham and Women's Hospital/Harvard Medical School, Boston, Massachusetts, United States of America; University Hospitals of Geneva, Switzerland

## Abstract

**Aims:**

To investigate the perceptions and reported practices of mental health hospital staff using national hospital electronic health records (EHRs) in order to inform future implementations, particularly in acute mental health settings.

**Methods:**

Thematic analysis of interviews with a wide range of clinical, information technology (IT), managerial and other staff at two early adopter mental health National Health Service (NHS) hospitals in London, UK, implementing national EHRs.

**Results:**

We analysed 33 interviews. We first sought out examples of workarounds, such as delayed data entry, entering data in wrong places and individuals using the EHR while logged in as a colleague, then identified possible reasons for the reported workarounds. Our analysis identified four main categories of factors contributing to workarounds (i.e., operational, cultural, organisational and technical). Operational factors included poor system integration with existing workflows and the system not meeting users' perceived needs. Cultural factors involved users' competence with IT and resistance to change. Organisational factors referred to insufficient organisational resources and training, while technical factors included inadequate local technical infrastructure. Many of these factors, such as integrating the EHR system with day-to-day operational processes, staff training and adequate local IT infrastructure, were likely to apply to system implementations in various settings, but we also identified factors that related particularly to implementing EHRs in mental health hospitals, for example: EHR system incompatibility with IT systems used by mental health–related sectors, notably social services; the EHR system lacking specific, mental health functionalities and options; and clinicians feeling unable to use computers while attending to distressed psychiatric patients.

**Conclusions:**

A better conceptual model of reasons for workarounds should help with designing, and supporting the implementation and adoption of, EHRs for use in hospital mental health settings.

## Introduction

The use of electronic health records (EHRs) has been increasing in anticipation of benefits such as reducing the rates of medical error, improving the quality of healthcare delivery, increasing staff productivity and reducing the costs of healthcare.[1]–[4] However, there has to date been limited evidence to support the claims made by proponents of EHRs, at least in the short-term. Also noteworthy is the growing appreciation that introducing an EHR system may result in new risks and other unintended consequences.[5], [6] For example, studies have reported patient-doctor communication during the clinical encounter can be negatively affected by the introduction of EHR systems.[7]–[9] Psychiatric patients may be more greatly affected than patients in other specialties as communication skills and confidentiality can be especially important in the relationships between these patients and healthcare professionals.[10]

The EHR potentially plays an important role in communication. Psychiatric patients often continue to be seen by multiple healthcare professionals (such as psychiatrists, psychologists, mental health social workers and family doctors) after hospital discharge, and cognitive, behavioural or emotional difficulties can limit a patient's ability to communicate important information to multiple people.[11] While the use of EHRs could help improve care co-ordination among different care providers, clinicians face the challenge of balancing information sharing with concerns about privacy and patient confidentiality. Social acceptance of past life events and the stigma still attached to mental illness remain significant concerns among those with mental illnesses, and worries about who can access digital health record information could lead to incomplete disclosure from patients to healthcare providers.[12]

Concerns about the use of EHR information are not limited to patients; psychiatrists were found to be the least likely to use EHRs among outpatient doctors from more than 14 medical specialties.[13] Salomon et al. reported that mental health professionals could be less willing to record highly confidential information in an EHR system than in a paper-based record, with respondents in that study reporting choosing generic wording for EHR data entry.[14] For example, details of childhood incest might simply be recorded as “inappropriate contact”. Some respondents also reported that they would keep a shadow record (i.e., their own record) for psychotherapy process notes because they were concerned that non-mental health care providers might misuse diagnostic terms for psychiatric conditions.

In a seminal paper, Gasser described how employees tried to integrate the use of computing into their daily work, including by altering their work practices to work around obstacles to achieving tasks, i.e., deliberately using the computer system in ways other than it was intended it should be used in order to get work done.[15] Subsequently, there has been considerable research into the use of ‘workarounds’ both within healthcare [16], [17] and in other sectors.[18]–[21]

For the purposes of our study, workarounds were defined as informal practices used to overcome workflow blocks.[22]–[24]

A conceptual model categorising reasons for workarounds related to healthcare IT, and particularly in the context of mental health hospitals, would be a valuable tool for future research. Understanding how staff use EHRs for psychiatric patients and why they use them in the ways they do could inform the development of EHR systems for mental health and, potentially, facilitate their acceptance and adoption by mental health hospital staff. The study reported here aimed to advance an understanding of the perceptions and reported practices of EHR users in mental health hospitals, and thereby to inform future implementations of EHRs in mental health settings. Our objectives were to identify specific examples of workarounds reported by hospital staff and possible reasons contributing to the workarounds; and to explore how the findings of our study compared with previous classifications of reasons for workarounds relating to the implementation of IT systems in order to develop a conceptual model for mental health EHRs.

## Methods

We undertook a qualitative, secondary analysis of semi-structured interview transcripts.[25]–[27] The semi-structured interview data we used were part of a larger dataset that was collected and analysed for a national, independent, longitudinal evaluation of the implementation of EHRs in secondary care in England.[28], [29]

The national evaluation [28] sampled hospitals that were among the first to implement centrally-procured EHR systems as part of a government programme to modernise the NHS in England.[30] Purposive sampling [31] was used to select and recruit a range of hospitals to give a heterogeneous sample with regard to hospital characteristics – such as the size of the hospital, its location and the type of care it provided – the EHR application that was then being implemented through a government programme (i.e., Lorenzo, RiO or Cerner Millennium); and the stage of the implementation. Each recruited site (n = 12) was treated as an individual case study,[32] with subsequent further analysis across sites.

The main source of data for the national evaluation was semi-structured interviews with a wide range of purposively selected hospital staff. Additional contextual interviews were conducted outside the case study sites. On average, these interviews lasted for approximately one hour, and they explored the interviewee's expectations, experiences and opinions of EHRs. Generic interview topic guides were adapted to particular groups of interviewees (e.g., IT personnel or healthcare practitioners) and the interview guides were further tailored in response to the evaluation team's iterative approach to data collection and analysis. Case study researchers also collected and reviewed documentary evidence and undertook on-site observations. Data collection was terminated at these case study sites when the evaluation team felt that data saturation had been achieved.[33] The data were analysed thematically.[34]

### Ethics

The original national study was classified as a service evaluation. Participation by interviewees was voluntary, written informed consent was obtained, and no compensation was given to participants directly. Level 1 Ethical Review by the Research Ethics Committee in the Centre for Population Health Sciences at The University of Edinburgh was obtained for the secondary analysis of data in this study.

### Sampling

Three of the 12 case study sites in the national evaluation were mental health hospitals. Of these three, one had implemented an EHR application that was being developed by the supplier for hospitals in the north, midlands and east of England, and two – both in London – had implemented RiO, a commercial, web-based EHR application supplied by CSE Healthcare Systems. RiO was the most widely implemented, centrally-procured mental health IT system in England. Having two London-based hospitals with RiO allowed us to combine the data collected from two sites for the purposes of the present study. We therefore chose the interview transcripts from these two hospitals as our sample for secondary analysis. The first of these sites, Hospital G, was a small-scale case study, which focused on the perspectives of senior clinicians and implementation team members who were actively involved in bringing RiO into their hospitals. Hospital M was an in-depth case study, with interviews with senior business and IT managers, the local implementation team and a range of healthcare practitioners, including nurses, psychiatrists, social workers and allied health professionals. Data were collected from the two hospitals between May 2009 and November 2010.

### Data collection and handling

The dataset for the present study consisted of professionally transcribed, verbatim transcripts of interviews with staff at hospitals G and M. We selected interview data that shed light on the focus of our investigation, namely EHR-related workarounds as reported by hospital staff. All transcripts had previously been checked and cleansed of identifying personal details by the national evaluation's researchers.

### Data analysis

The interview transcripts were read repeatedly by the researcher (GS) who, in conjunction with the co-authors (AS and AR), identified all the transcript passages that referred to workarounds and possible reasons for workarounds. The data relating to workarounds were then analysed thematically, without imposing any prior coding categories. Thematic analysis is a method for identifying and reporting themes (patterns) in qualitative data and helps in the organisation and description of data. Thematic analysis is a flexible qualitative approach to seeking repeated patterns across a data set, which nonetheless follows a series of steps, i.e., familiarisation with the transcribed data set; generating initial codes from relevant segments of the data; collating the codes under broader, thematic headings; reviewing the initial organisation of themes; and then describing and interpreting the meaning in those themes. [34] We used QSR NVivo Version 9 software to aid managing the data and coding the dataset.

## Results

We analysed 33 transcripts of interviews with staff at mental health hospitals G and M. Hospital G was one of the first to implement a basic version of RiO (Version 4) and it later upgraded to Version 5.1. Version 5.1 used card-controlled (Smartcard) access as a security feature and introduced connectivity with the central NHS database and messaging service, so giving authorised users access to the personal demographic information of NHS patients. Hospital M initially took Version 5.1 of RiO.

Examples of workarounds that were mentioned by these interviewees were: delayed or retrospective entering of data; doctors dictating notes and leaving data entry to administrative staff; entering information in the wrong place in the EHR; and using the EHR while it was logged in under another user. After identifying the examples of workarounds in the transcripts, we identified possible reasons for workarounds, which we organised under four main category headings: operational; cultural; organisational; and technical factors (Table 1).Below, we present the identified workarounds followed, in turn, by the four categories we identified of possible reasons, with supporting quotes from the transcripts given below.

**Table 1:** The four main categories of possible reasons for identified workarounds with the themes and sub-themes and, in brackets, the number of times each was mentioned in the interview transcript dataset.

**Operational factors** (81 mentions)
Theme 1. Integration with work practices
○ Aspects of EHR design not suitable for mental health settings (12)
○ Users' work practices and system requirements not aligned (10)
○ Unsuitable for some consultations (2)
○ System structure for data entry not clear/user-friendly (16)
○ Data confidentiality concerns (9)
Theme 2. System does not meet different users' needs
○ Lack of certain mental health-related functionalities (8)
○ Lack of integration with IT systems of different mental health and other care providers (4)
○ System not adequate for reporting purposes (12)
Theme 3. Contract-related issues
○ Frustration with lack of local configurability and customisation (4)
○ Frustration with slow change process (4)
**Cultural factors** (19 mentions)
Theme 1. Cultural change
○ IT competency among staff (11)
○ Age and users' comfort with IT (2)
○ Anxiety about or resistance to change (5)
○ Lack of enthusiasm for the system (1)
**Organisational factors** (31 mentions)
Theme 1. Communication of EHR vision
○ Insufficient user engagement with EHR (4)
○ Lack of standardised use of the system (4)
Theme 2. Resources in the hospital
○ Lack of resources (e.g., inadequate provision of terminals) (10)
○ Training criticisms (e.g., intensity, timing, appropriateness) (13)
**Technical factors** (19 mentions)
Theme 1. System and supporting infrastructure
○ System instability (10)
○ Computers slow (5)
○ Infrastructure problems (4)

### Examples of workarounds

Interviewees reported that rather than entering information into the EHR system immediately, they would often update the information on the patient some hours, or even a few days, later.

Delays with putting patient information into the system could also occur because doctors sometimes dictated notes and left data entry to administrative staff.

Interviewees also spoke about entering information in the wrong place in the EHR, in particular, using a general section for notes in the EHR system for most data entry. Staff, however, recognised that not conforming to the structure of the EHR would have negative consequences in the future, including making it difficult or impossible for care providers to retrieve relevant information from the system at a later date.

Although every member of staff who used the EHR system was required to log in to the system using their own Smartcard and user name, it was acknowledged that some staff might use computer terminals while they were logged-in under another user, despite this being “totally illegal”.

### Possible reasons for workarounds

Potential contributing factors to workarounds identified in the transcripts were classified into four broad themes: operational, cultural, organisational and technical factors.

#### Operational factors

Operational factors related to the day-to-day work carried out by mental health professionals and other hospital staff (Box 1). Many interviewees were of the view that the EHR system did not integrate well with their existing work practices and required more time of them to use.

“*I don't think it's the IT that is the problem, I think it's whoever has designed the system with making assumptions about how our work is organised that doesn't really fit with our work – that's the difficulty.*” **Mental health social worker.**

“*There are many different teams within the mental health system and you need to take all of that into consideration. I think it feels like there is one model of care and everyone needs to kind of adapt to that.*” **Crisis team social worker.**

The EHR system was perceived as being medically-oriented and more suitable for acute settings where patients were typically treated and then discharged. Some interviewees reported feeling restricted by the options available in the EHR in the context of delivering a mental health service with on-going support for psychiatric patients, who often also had physical co-morbidities. Others reportedly found it difficult to know where to enter certain information or which “box to tick”.

“*It feels as though there is an assumption of through-put that people are going to come and have a diagnosis and have a treatment and be discharged. Mental Health Services don't really work like that. … It feels as though the system has expectations that are not realistic.*” **Mental health social worker.**

It was said that, if staff wanted to be able to leave work on time, they had to spend less time with patients so that they could input information into the EHR system. An interviewee articulated that this had negative consequences for patient care, and particularly for caring for mental health patients with whom it could take years to build up trust in a therapeutic relationship. By spending less time with psychiatric patients, the quality of those therapeutic relationships was thought likely to suffer.

“*I think there are some things I haven't written. I mean, things that clients have told me that have been very confidential that I might have put in hand written notes, in paper notes…*” **Mental health social worker.**

The information stored in an EHR might also be less complete than in a paper record. Interviewees raised concerns about confidentiality and also about the practicality of using the EHR system while seeing highly unsettled patients.

“*They are not going to be able to do it while they are with the patient, because of issues like risk. These are patients that are really quite disturbed. You can't kind of be faffing around getting them by computers.*” **Consultant psychiatrist.**

Importantly, the EHR system at that time was perceived not to meet clinical needs with regard to regulations specifically relating to mental health, including national mental health legislation.[35] Interviewees reported that there were functions that they needed to use in their daily work to meet legislative requirements but were not, at the time of data collection, available in the hospitals' systems.

Interviewees expressed the wish for a greater ability to customise the system to tailor it to their own requirements. As part of the contractual arrangements at the time, hospitals implementing new EHR systems under a government NHS modernisation programme only indirectly liaised with the system suppliers via a Local Service Provider.[28], [29] Interviewees who complained about being unable to tailor the system locally felt this was a result of the contractual terms under the government programme, rather than necessarily due to limitations inherent in the EHR system.

“*You can't delete out the bits that aren't relevant. So you would have the whole document, which includes things like forensic history and murder, manslaughter, arson, which are perhaps not appropriate to an elderly person presenting for the first time to Mental Health Services with some mild memory problems.*” **Geriatric psychiatrist.**

Importantly, concerns were raised by these interviewees about the lack of integration between the hospital EHR system and other IT systems used locally by, for example, NHS psychologists and local authority social workers who were also involved in the care of their patients. It was seen as crucial that mental health IT systems should be able to link with local authority IT systems because caring for people with mental health problems often involved liaising with other organisations in the community.

Other interviewees expressed disappointment in the EHR system's suitability for reporting performance data. Some acknowledged that it was difficult for a mental health hospital system to be designed to be clinically useful and easy for staff to use and yet also to allow for easy data extraction to run reports for statistical and operational management purposes.

“*…for Substance Misuse Services it is not delivering key performance data that we have to give to national sources in order to prove that we are performing. Again, we are having to find workarounds for that, which really should be in there from day one.*” **Operational director of services.**

#### Cultural factors

Many interviewees perceived that the change from paper records to EHRs involved a big cultural change for staff, especially for those who were less familiar with using IT. Interviewees acknowledged that staff who were not familiar with computers and who lacked IT-related skills struggled with moving to EHRs. This could lead to spending less time with patients and more time on computers, and it was thought to contribute to staff anxieties about using the new system.

“*From my point of view, it was very much around the preparing for that complete shift in culture, around the move from paper records to electronic, really. I mean, obviously, a lot of our staff couldn't even use a PC [personal computer]. They weren't even accessing emails…*” **Director of services.**

“*Myself and colleagues of my generation who had to learn computers in later life I think have found it difficult.*” **Mental health social worker.**

An interviewee raised the concern that some members of staff had physical problems, such as arthritis, and were therefore unable easily to type. Older age was suggested as a factor in users' general comfort with using IT systems and with individuals' levels of basic IT-related skills such as typing competence.

Reported low levels of IT literacy among hospital employees were also thought to be related to staff “resisting” change to new EHR systems.

“*We weren't quite prepared for the kind of mental impact on morale and how staff found it very difficult… because people, in our field of work, aren't generally IT literate. They don't have that interest. There is a lot of resistance to change.*” **Community mental health nurse.**

#### Organisational factors

Organisational factors related to the senior management level in the hospital and in the NHS more widely. Some interviewees felt that hospital leaders did not understand the views of the mental health clinicians, who were the end-users of the system. Other interviewees perceived that hospital managers had not done enough to communicate successfully to their staff what the government's healthcare modernisation programme was about and how implementing EHR systems fitted into a wider vision of improving patient care and efficiency in the NHS.

“*It's getting the users to understand and the hospital to understand that the system is theirs and they own it. It's up to them to get the best out of it. The danger is they feel it's thrust on them and they have to use it. You need to change that perception.*” **Project manager.**

Lack of hospital resources frequently came up in the interviews. For example, interviewees reported there were not enough computers available for staff to use when they needed them and that they would often have to wait to use a computer, especially during handover periods. The lack of physical space to place computers was also mentioned. Not having enough working computers available was suggested as a reason for staff continuing to use paper.

Users of the new EHR system were also critical of the training they had received. Some reported that the time between their training and implementing the new EHR system was too long with interviewees admitting they had forgotten what they had learnt during the training by the time they started to use the system. Others reported they found much of the training to be irrelevant to their specific professional role. It was felt training sessions should be tailored much more closely to the different needs of various users of the system, allowing for more thorough and detailed training. This required tailoring training not just to different user groups, such as doctors and nurses, but to different staff roles within those groups, for example, different groups of nurses.

“*If you are a doctor you've got a certain type of training. If you were an admin person, you've got a certain type of training. But if you are a nurse and you work in an inpatient ward, it's very different to what you do if you are a nurse and you work in a community team. And so, you had people sitting in training session where maybe 50% of what was being trained was not really applicable to their job. And then, the bit that was applicable to their job as a consequence, you didn't get the in-depth detail of maybe, if you could just spend your whole training session just on that bit that applied to you, you could have got into much more detail and sort of more clarity.*” **Business manager.**

#### Technical factors

Technical factors included issues related to the existing technical infrastructure in the hospitals. Reports of system instability were common. Interviewees reported that the EHR system “crashed” quite often and could be down for extended periods of time.Reluctance by some staff to use the new IT system was attributed to the system's instability.

“*So we had teams saying, we are not putting our risk assessments on the system because we think it's too risky. The system goes down and it's not available.*” **Hospital manager.**

Keeping a paper back-up copy of certain information was therefore seen by some as a necessity for times when the EHR system was unavailable. Others reported finding the IT systems slow to use, with log-in times too long.

“*Even if it's just logging on, it's quite slow.*” **Occupational therapist.**

These technical problems were often attributed to the supporting infrastructure in the hospital rather than to the EHR system itself.

“*It just crashes. So, every time we report this to the help desk that is in the hospital, we know by now that it's nothing to do with the help desk, they can't help us because it's something to do with some connection or something to do with the server.*” **Crisis team staff member.**

## Discussion

### Main findings

We found several examples of workarounds following the implementation of an EHR system in two mental health hospitals, these including: delayed or retrospective entering of data; doctors leaving data entry to administrative staff; entering information in the wrong place in the EHR; and using the EHR while it was logged in under another user – all of which have the potential to compromise the quality and safety of care. Through our thematic analysis we were able to code the staff-identified factors contributing to these workarounds into four categories: operational, cultural, organisational and technical. Operational factors were predominant in this study and included users' perceptions that the EHR did not integrate well with existing work practices, so interrupting workflows and requiring additional staff time to be spent on computers. Several of these operational factors were quite specific to the context of a mental health hospital, for example, the immediate demands of managing unsettled patients being incompatible with finding and using a computer, and diverse professional groups involved in caring for mental health patients in hospital attempting to adapt to an EHR system that was perceived to be better oriented to acute medical care. Cultural factors concerned the shift from paper-based records to an EHR and our findings highlighted low levels of IT skills among some hospital staff, including lack of basic typing skills. Organisational factors included lack of appropriately specific training for diverse job roles, limited hospital resources that resulted in inadequate numbers of computers for staff to use and challenges of successfully communicating to staff the wider “vision” of improving healthcare through using EHRs. Technical factors particularly related to the local infrastructure to support these hospitals' new IT systems.

### Considering this study in the context of the wider literature

It has previously been noted that where IT systems enforce procedures that are at odds with effective work practices, employees might resort to workarounds such as retrospective data entry.[21] It is likely that the interviewees in this study delayed data entry so as to manage their workloads and to deliver direct patient care in a timely manner. Similarly, the workaround of using the EHR while it was logged-in under another user could be attributed to individuals trying to manage their workflow, with users indicating they found it too time consuming to log-out and to log-in again using their own user names.

The workaround of doctors leaving data entry to administrative staff may have resulted from a combination of factors in addition to managing workflow, such as resistance to changing practice to adapt to digital working and perceptions that the EHR system was not user-friendly. Clinicians may be more reluctant to switch from a paper-based system to an EHR if they have poor typing skills or if they prefer to write longer, free text notes instead of more concise entries to fit into the IT system.[36] EHR systems usually require users to enter data in structured formats for searching, reporting or managerial purposes but such formats may require clinical users to spend more time on entering the data.[37]

Entering information in the wrong place has previously been identified in the literature on workarounds involving Computerised Physician Order Entry (CPOE) systems.[38] Those authors argued that when the CPOE system interface required users to navigate through multiple screens to get to the correct place for entering information, busy clinicians might select the ‘miscellaneous’ section rather than spend time looking for the right section. Another possible contributing factor for this workaround is users not always knowing where information should be entered in the EHR, perhaps related to inadequate initial training or too long a delay between training and working with the live system.

The socio-technical perspective – recognising the interplay between an organisation's social and technical systems – provides an overarching framework for understanding workarounds and the reasons underlying them. [39] When healthcare IT systems do not integrate well with existing work processes and practices, users struggle with a system that does not fully support them to do their work and they develop workarounds in order to live with the IT system while avoiding system demands that are perceived to be unrealistic.[40], [37]

Nonetheless, several possible contributing factors to workarounds identified in this study applied particularly to the use of an EHR system in the mental health setting. For example, having outcome options such as discharge or admission to hospital, rather than on-going support from a variety of agencies, suggests that EHR systems for mental health settings need to take greater account of how caring for mental health patients differs from patient care in other healthcare settings. It was also clear from these interviewees' accounts that in order to maximise the potential benefits of having a mental health EHR system, that system must integrate with IT systems used by others involved in the care of mental health patients inside the hospital and beyond, and particularly with IT systems used by social services.

Further, our study highlights how the nature of some staff-patient interactions in mental health hospitals is likely to pose particular challenges to always using an EHR system. For example, using a computer during interactions with patients who were highly distressed or agitated raised issues of risk, and retrospective entering of information might always be seen as a necessity in these situations.

### Frameworks for analysing possible reasons for workarounds

Koppel et al.[17] studied workarounds in a healthcare setting involving the use of Bar Code Medication Administration (BCMA) systems in hospitals and classified the probable causes of workarounds into five categories: technology-related; task-related; organisational causes; patient-related; and environmental causes. Technology-related causes related to the BCMA software or hardware, including difficult-to-navigate screens, while task-related causes included issues such as users' perception that using the BCMA would slow down work processes. The predominant category of operational factors in our study could therefore be seen as a combination of Koppel et al.'s technology-related and task-related categories, although our data analysis generated a discrete category for technical factors, particularly relating to infrastructure. Organisational causes included users having inadequate training in the use of BCMA, similar to findings in our study under organisational factors, and such issues as hospital policies being incompatible with the use of BCMA and users not understanding the role of the BCMA in patient safety.

In Koppel et al., patient-related causes concerned patients' special circumstances which resulted in the BCMA not being used, for example, if patients had brought medications from home, these were not always barcoded and scanned. Lastly, environmental causes comprised factors related to the hospital's physical structure and of the location of related technologies, for instance, certain areas in a hospital not having wireless BCMA connectivity.

In contrast to our study Koppel et al. found that organisational causes were associated with most of their identified workarounds. Cultural factors in our study, such as users being uncomfortable with using unfamiliar technology and staff anxieties about adapting to new ways of working, were not highlighted in the study by Koppel et al.

Although also investigating possible causes for technology workarounds in hospitals, the BCMA study focused on a particular staff group involved in a specific hospital-based task, i.e., medicines administration; the differences between the findings of that study and this, notably the predominance in our study of day-to-day operational factors rather than organisational factors, likely reflect the different scale and scope of the hospital technology under study, the wide range of disparate staff groups who use EHR systems and, given many of our operational factors were specific to mental health care provision, the particularities of a mental health hospital context.

Also seeking to understand reasons for non-compliance with the intended use of IT systems, Sobreperez reported three categories relating to technology use in which perceptions of the IT system varied between different users.[21] These categories may serve as a more useful foundation from which to conceptualise workaround reasons in the context of healthcare IT implementations and specifically EHR implementations in mental health settings. Findings from our study can be compared with the framework proposed by Sobreperez and suggest how that framework might be adapted to serve future research into mental health hospital EHRs.

The first category proposed by Sobreperez of “proceduralisation”, which referred to IT systems demanding procedures that were perceived to go against effective work practices, corresponds well to the category of operational factors in the present study. The second category proposed by Sobreperez, “acceptance”, which included users avoiding using the new technology, maps to the category of cultural factors in our study, although our cultural category is broader to encompass issues such as users' general IT skills.

The third category proposed by Sobreperez was “culture and control”, which referred to organisational culture and management control. This could be broadened to include those factors that were categorised in this study as organisational factors. Our fourth category of technical factors has no corresponding category in the three-category framework proposed by Sobreperez. A fourth category of technical factors could therefore be usefully added to the Sobreperez framework to analyse possible reasons for workarounds related to the implementation of EHR systems in mental health settings. This underscores the importance of the technical dimension and the interplay between it and social dimensions in producing workarounds. Table 2 illustrates the relationship between the category framework proposed by Sobreperez [21] and the one derived from findings from the present study.

**Table 2:** Framework for understanding reasons for mental health Electronic Health Record (EHR) workarounds.

Categories identified by Sobreperez	Corresponding and one additional category identified in our study
Proceduralisation	Operational factors
Acceptance	Cultural factors
Culture and Control	Organisational factors
	Technical factors

The workaround category framework by Sobreperez [21] as it maps on to the framework derived from our qualitative study of reasons for mental health EHR workarounds, where we identified an additional, fourth category of technical factors.

### Implications for policy and practice

If EHR systems are to be used effectively in hospitals and their hoped-for benefits realised, the reasons for staff workarounds, with their potential for adverse consequences, need to be identified and addressed. As day-to-day operational factors made up the largest group of possible reasons for workarounds identified in our study in mental health hospitals, it is clearly important that real-life clinical workflows and the design, functions, and availability of the EHR system be better reconciled, as has previously been emphasised by other authors.[41].

Policy has shifted since the demise of the government's centralised programme to implement standardised EHR systems in hospitals in England. Policy now is for NHS organisations to invest in a range of locally chosen solutions within a framework of national standards. Integrating the range of hospital IT systems such that digital information can be shared across the hospital and, finally, further afield with other organisations is key to current policy. While full interoperability across boundaries is conceptualised as the last step of an incremental maturation of a digital NHS, policy makers, and system suppliers, need to be aware that this is a more immediate concern among those in the mental health sector; the medical model of care for hospital patients differs from the complex, shared delivery of long-term care for mental illness. The further challenge, apparent from our study, is to balance widely accessible digital information to support healthcare delivery in mental health hospitals with addressing concerns about possible adverse consequences of sharing information in the context of psychiatric care.

### Strengths and limitations

This research drew on multiple interviews conducted by experienced qualitative researchers with a wide range of hospital participants. By focusing on a selected sub-set of previously collected data, we were able to identify similarities between EHR workarounds in mental health hospitals and workarounds in other settings while also highlighting areas where using an EHR system posed challenges particular to mental health settings. By comparing previously identified categories of factors that contribute to IT-related workarounds with categories that were derived from analysing our data, without prior coding themes, we have been able to offer a revised framework of categories to inform the design of future EHRs for use in mental health settings and to inform the implementation strategies employed.

Study limitations need however to be considered. The study drew on data that were gathered at a point in time in a rapidly evolving landscape of development of healthcare IT systems, NHS hospitals' resources and government policy. We relied on secondary analysis of a single source of data – interview transcripts. These interviews had not been conducted for the specific purpose of exploring mental health EHR-related workarounds but rather to explore experiences and views of EHR implementations in hospitals more widely. Hence, it is possible that there were other examples of workarounds, and more information on contributing reasons workarounds, that were not mentioned in these interviews. It is not possible to state that data “saturation” had been reached with respect to the specific research questions of the secondary analysis study. Similarly, the researcher undertaking secondary analysis of the data was unable to use interaction cues during interviewing to enhance the understanding of the interviewees' meaning, or to go back to individual interviewees to check her interpretation of the written texts available to her. In secondary analysis of data, it is not possible to conduct data collection and analysis iteratively as would often be done in primary qualitative research (and was done in the original study). Nonetheless, the researcher was working very closely with members of the team who had originally generated and analysed this dataset. Finally, this study focused on a single EHR system, at early stages of being implemented in two hospitals, and the findings may not extend to other mental health EHR systems or to other, later time points in the implementation process.

## Conclusions

Most of the possible, contributing factors for workarounds identified in this study were associated with operational factors that directly affected users of the system, such as the EHR system being perceived as taking too much time to use. Several of these operational factors seemed to relate specifically to EHRs in mental health settings, notably the lack of certain options and functionalities that mental health staff required for their daily work and the system not being integrated with the IT systems of other providers of care for mental health patients. Other issues, such as IT skills levels among hospital staff, the adequacy of training for using the new IT system, and the numbers of computers available for users, also featured in our findings. This suggests system suppliers and mental health organisations that plan to implement an EHR system need to pay extra attention to these considerations to increase system acceptance by clinicians and other staff. In particular, issues specific to mental health contexts need to be better accommodated if EHR implementations in mental health hospitals are to be enhanced. Further research is now needed to assess the reliability of these findings and our proposed four-category framework of reasons for workarounds in other mental health settings and implementation contexts following the demise of the government's centralised EHR programme, and involving different mental health EHR systems.
